# Increased Expression of microRNA-141-3p Improves Necrotizing Enterocolitis of Neonates Through Targeting MNX1

**DOI:** 10.3389/fped.2020.00385

**Published:** 2020-07-28

**Authors:** Hui Chen, Lichun Zeng, Wei Zheng, Xiaoli Li, Baixing Lin

**Affiliations:** Department of Neonatology, Shenzhen Hospital of Southern Medical University, Shenzhen, China

**Keywords:** necrotizing enterocolitis, microRNA-141-3p, motor neuron and pancreas homeobox 1, cell viability, apoptosis

## Abstract

**Objective:** MicroRNA-141-3p (miR-141-3p) has been investigated in various kinds of cancers. This research delves into the functions and regulatory mechanisms of miR-141-3p in necrotizing enterocolitis (NEC) of neonates.

**Methods:** NEC tissues were obtained from neonatal mice, and subsequently, expression of miR-141-3p and motor neuron and pancreas homeobox 1 (MNX1) was assayed via RT-qPCR. Moreover, the intestinal histopathological changes and histiocytic apoptosis were observed via hematoxylin and eosin (H&E) and TUNEL staining. The correlative inflammatory factors and oxidative stress markers were evaluated to uncover the influence of miR-141-3p in NEC tissue damage. Further, the relation between MNX1 and miR-141-3p was predicated, and the functions of MNX1 in inflammatory response and cell growth of IEC-6 cells were investigated.

**Results:** Downregulated miR-141-3p and upregulated MNX1 were discovered in NEC tissues. Moreover, miR-141-3p clearly alleviated inflammation response and oxidative stress damage in NEC, which was achieved through regulating inflammatory cytokines (IL-1β, IL-6, and TNF-α) and oxidative stress markers (MPO, MDA, and SOD) expression. MNX1 was forecasted as a target gene of miR-141-3p; meanwhile, MNX1 overexpression overturned the influence of miR-141-3p in the inflammatory response and cell growth process of IEC-6 cells.

**Conclusion:** These explorations reveal that increased expression of miR-141-3p could improve the damage to intestinal tissues in NEC through targeting MNX1. The research might exhibit a neoteric therapeutic strategy for NEC.

## Introduction

Necrotizing enterocolitis (NEC) is an acquired illness, which is the most familiar neonatal gastrointestinal emergency, and it is also a dominating reason for intestinal perforation and systemic inflammatory response syndrome (SIRS) in neonates (1, 2). NEC affects digestive system function, and severe cases can cause systemic symptoms, such as shock, acidosis, and jaundice (3, 4). The representative symptoms of NEC in the clinic incorporate abdominal distension, vomiting, diarrhea, and bloody stools (5). Adequate parenteral nutrition; maintaining the balance of water, electrolyte, acid, and base; and nutritional support are the treatment policy of NEC (6). Although there are advances in neonatal nursing, NEC is still a leading cause of morbidity and mortality in premature infants (7). Thus, to clarify the pathogenesis of NEC and explore its possible diagnostic and therapeutic targets will be beneficial to the clinical therapy of this disease.

MicroRNA (miRNA) is one kind of endogenetic RNA molecule with a length of ~22 nucleotides, which joins in post-transcriptional gene expression in plants and animals (8). Recently, a correlative report disclosed the momentous roles of miRNAs in NEC (9). Among these, the importance of miR-21 has been testified to regulate the inflammatory responses in NEC (9). Additionally, the dysregulation of miR-431 was discovered in intestinal tissues of infants with NEC, which might participate in reinforcing the inflammatory response in NEC tissues and be conducive to pro-inflammatory pathophysiology (10). MiR-141-3p is an extensively researched miRNA, which has been investigated in various cancers (11, 12). Shen et al. reveals that miR-141-3p could allay chronic inflammatory pain through suppressing HMGB1 *in vitro* and *in vivo* (13). Nevertheless, relevant research about the influences of miR-141-3p in NEC remains unreported.

Motor neuron and pancreas homeobox 1 (MNX1) is also named Hb9 or Hlxb9, which can encode transcription factor (14). MNX1 was ascertained to be a newfangled reason for permanent neonatal diabetes in a kindred study (15). Moreover, one research illustrates that upregulated MNX1 has been discovered in prostate cancer cells, which might be an innovative biomarker of this cancer (16). Other research also uncovers that abnormally expressed MNX1 is linked to infantile acute myelocytic leukemia (AML) (17). But the effect and the potential regulated mechanisms of MNX1 are still indistinct in NEC of neonates.

In our research, we discovered the ectopic expression of miR-141-3p and MNX1 in NEC tissues. In addition, miR-141-3p improved the inflammation response and oxidative stress damage in NEC through targeting MNX1. Outside of these, MNX1 joined in modulating the influences of miR-141-3p in IEC-6 cell vitality and apoptosis. These explorations might offer a new therapeutic thought for NEC.

## Methods and Methods

### NEC Model Construction and Groups

Experimental male C57BL/6 mice (4–7 days) were achieved from Jiesi Experimental Animal Co., Ltd. (Shanghai, China). The mice were divided into four groups: Control, NEC, NEC+miR-141 NC, and NEC+miR-141 mimics. Breast-feeding and enema with normal saline was utilized to establish a Control group; artificial formula feeding combined with hypoxic cold stimulation was used to establish the NEC group; breast-feeding and enema with 0.2 mL miRNA NC was employed to build the NEC+miR-141 NC group; artificial formula feeding combined with hypoxic cold stimulation and enema with 0.2 mL miR-141-3p was used to structure the NEC+miR-141 mimics group. The disposed detailed procedures were performed according to previous research (18). After anesthetizing the mice with pentobarbital sodium, the intestinal tissues were collected and washed by normal saline. The obtained samples were stored in liquid nitrogen and placed in a −80°C refrigerator.

### Histopathologic Examination

Two centimeters of the terminal ileum were taken, which were then immediately fixed in 10% neutral formaldehyde as well as embedded in paraffin and prepared in 3 μm sections. Subsequently, the hematoxylin and eosin (H&E) staining was carried out to disclose the functions of miR-141-3p in NEC. Additionally, the intestinal histopathological score was judged on the basis of a report from (19). The highest score observed in the sample was taken to determine the degree of intestinal damage, and the histological score ≥2 points was determined as NEC.

### TUNEL Staining Procedure

Cell apoptosis from the NEC tissues was evaluated through utilizing the DeadEnd TM fluorometric TUNEL system kit (Promega, Madison, WI, USA). The detailed procedures refer to the previous research (20, 21).

### Cell Transfection

The experimental vectors of miR-141 mimics/mimics NC and pcDNA-MNX1/pcDNA-NC were established by GenePharma (Shanghai, China). On the basis of the lipofectamine 3000 reagent (Invitrogen), a cell transfection assay was carried out for 48 h.

### Evaluation of Inflammatory Cytokine Levels

The paired ELISA kits of IL-6, IL-1β, and TNF-α (all from Shanghai Biotechnology Co., Ltd., Shanghai, China) were applied to evaluate the concentrations of IL-6, IL-1β, and TNF-α in intestinal tissues and IEC-6 cells after the corresponding vector transfection or LPS treatment. The optical density (OD) values of each well were measured by a microplate reader (Thermo Scientific Corporation, Massachusetts, USA) at 450 nm.

### RT-qPCR

Total RNA from intestinal tissues and IEC-6 cells was isolated with the aid of TRIzol reagent (Invitrogen). The cDNA template was compounded on the PCR-Cycler. The RT-qPCR procedure was implemented via applying ABI7500 quantitative PCR instrument (ABI, Foster, CA, USA). The CT values achieved from the experiment were analyzed through executing the 2^−ΔΔCt^ method (22). The primer sequences of IL-6, IL-1β, TNF-α, and GAPDH were synthesized by the Sangon Biotech (Shanghai, China) and are listed in Table 1.

**Table 1:** The correlative primer sequences for RT-qPCR.

**Name**	**Primer sequences (5^**′**^-3^**′**^)**
IL-6-F	GATACCACTCCCAACAGAC
IL-6-R	CTTTTCTCATTTCCACGAT
IL-1β-F	GTGGTGTGTGACGTTCCCATTA
IL-1β-R	CCGACAGCACGAGGCTTT
TNF-α-F	TGCAGCAGGACATCAAGTTC
TNF-α-R	TACGCCTCAGCAGTCTCCTT
GAPDH-F	GCTTCGGCAGCACATATACTAAAAT
GAPDH -R	CGCTTCACGAATTTGCGTGTCAT

### Detection of MPO, MDA, and SOD Levels

Distalileum tissue samples were achieved to prepare the tissue homogenate (10% w/v). Conforming to the description by Hillegass et al. (23), myeloperoxidase (MPO) activity was examined. Additionally, the levels of malondialdehyde (MDA) and superoxide dismutase (SOD) were measured according to the corresponding kit specifications (Jiancheng Bioengineering Institute, Nanjing, China).

### Luciferase Reporter Assay

The miR-141-3p fragment or the 3′-UTR of MNX1 was amplified by PCR, and subsequently, the MNX1-WT was structured by using the pmirGLO luciferase vector (Promega, Madison, WI, USA). MNX1-MUT was established through applying a site-directed mutagenesis kit (Stratagene, La Jolla, CA, USA). These above-involved vectors were co-transfected into IEC-6 cells with miR-141-3p mimics and mimics NC. The luciferase activity was evaluated on the basis of the dual luciferase reporter assay system (Promega) after transfection for 48 h.

### Western Blot Assay

After mimics NC and miR-141 mimics transfection, the proteins from IEC-6 cells were extracted in line with the protein extraction kit (Beyotime, Shanghai, China). The protein concentration was assessed by a BCA kit (Beyotime). The experimental steps of SDS-PAGE and PVDF membrane transfer were carried out according to the foregone research. After this, the membranes were blocked by BSA, and the primary antibody against MNX1 and the corresponding second antibody were utilized to co-culture with the PVDF membranes. The protein bands emerged via using the enhanced chemiluminescence (ECL) reagent (Amersham Biosciences Corp., Piscataway, NJ) and, meanwhile, quantified through Quantity One software (Bio-Rad, Hercules, CA, USA).

### Detection of Cell Viability

The intestinal epithelial cell line, IEC-6, was obtained from ATCC (Rockville, MD, USA), which was cultured in DMEM mixed with 10% FBS. After LPS irritation and the corresponding vector transfection, the disposed IEC-6 cells were collected and inoculated into 96-well plates. After 24 h culture, cells were reacted with 20 μL MTT solution (5 mg/mL, Sigma, St. Louis, MO, USA) for an extra 4 h. When terminating the incubation, 150 μL DMSO (4%, Sigma) was used to accelerate the crystal dissolution. After accomplishing these procedures, a microplate reader (BioTek, Winooski, VT, USA) was applied to test the absorbance at 570 nm.

### Determination of Cell Death

After LPS irritation and the corresponding vector transfection, an Annexin V-PI Kit (Invitrogen, Life Technologies, USA) was utilized for exploring the apoptosis ability of IEC-6 cells. The disposed IEC-6 cells were collected and, meanwhile, washed once with PBS (Invitrogen) as well as resuspended in 1 × binding buffer. Afterward, 5 μL Annexin V-FITC and 5 μL PI staining solutions were added into 400 μL cell suspension; in the meantime, cells were incubated for 30 min at 4°C under a dark environment. Immediately, the apoptotic proportion was monitored via executing a flow cytometer (BD Biosciences, Heidelberg, Germany) and analyzed by the FlowJo 7.6 software (Tree Star Inc., San Carlos, CA, USA). Furthermore, the level of lactate dehydrogenase (LDH) was examined by the paired kits (Beyotime, Shanghai, China).

### Statistical Analysis

Relying on GraphPad Prism 7.0 software (San Diego, CA, USA), the data were managed and statistical analyses conducted, and these data were presented as mean + SD. The one-way ANOVA accompanied by Tukey's multiple comparisons test was utilized to compare the differences in multi-groups. *P* < 0.05 manifested that the consequences possessed significant differences.

This study was conducted after obtaining approval of Shenzhen Hospital of Southern Medical University's ethical committee, and all experiments were in accordance with the guide for the care and use of laboratory animals established by the U.S. National Institutes of Health (Bethesda, MD, USA). All the animal experiments were conducted in the Science Building of Shenzhen Hospital of Southern Medical University.

## Results

### Abnormal Expression of miR-141-3p and MNX1 in NEC Tissues

**Figure 1:**
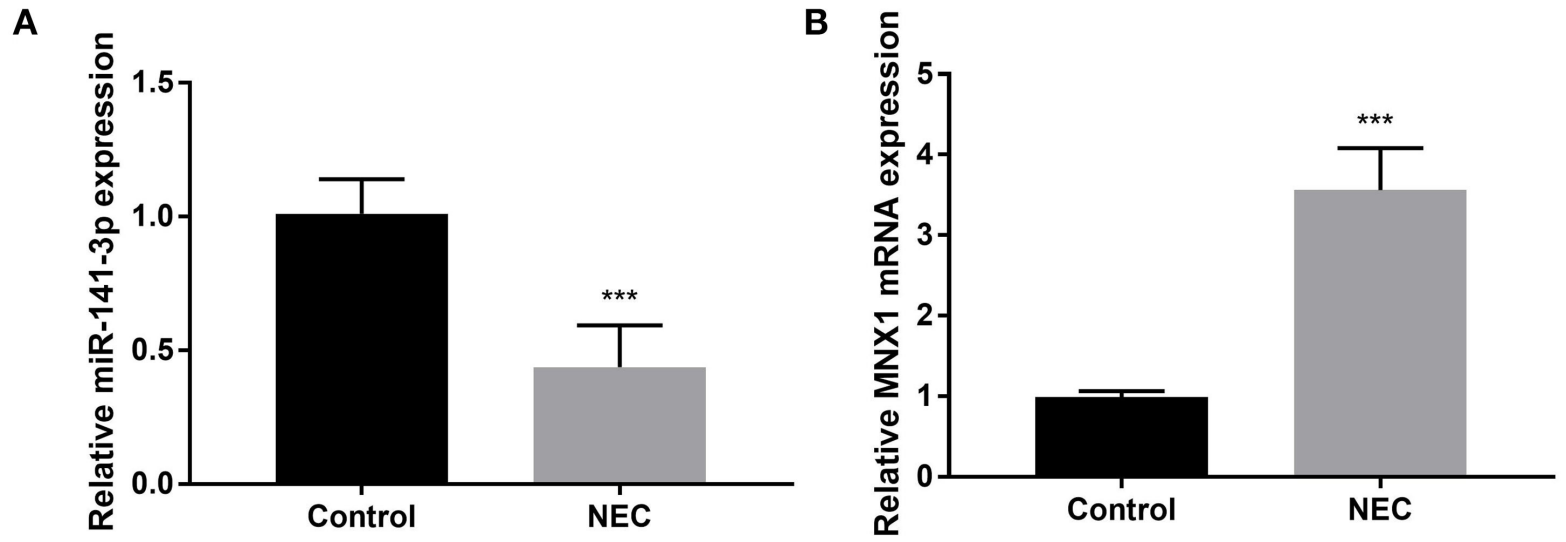
Aberrant expression of miR-141-3p and MNX1 in NEC tissues. NEC tissue was obtained from male C57BL/6 neonatal mice (*n* = 8), and expression of **(A)** miR-141-3p and **(B)** MNX1 in these tissues was tested via RT-qPCR. ***P < 0.001 in comparison with the Control group.

The NEC tissues obtained from eight male C57BL/6 neonatal mice were used in the experiment; thereafter, the RT-qPCR assay was carried out to examine miR-141-3p and MNX1 expression in NEC tissues. We noted that miR-141-3p was significantly down-expressed in NEC tissues in comparison with that in the control group (*P* < 0.001, Figure 1A). On the contrary, up-expressed MNX1 was observed in NEC tissues (*P* < 0.001, Figure 1B). These consequences revealed the aberrant expression of miR-141-3p and MNX1 in NEC tissues.

### miR-141-3p Mitigated the Damage to Intestinal Tissues in NEC

**Figure 2:**
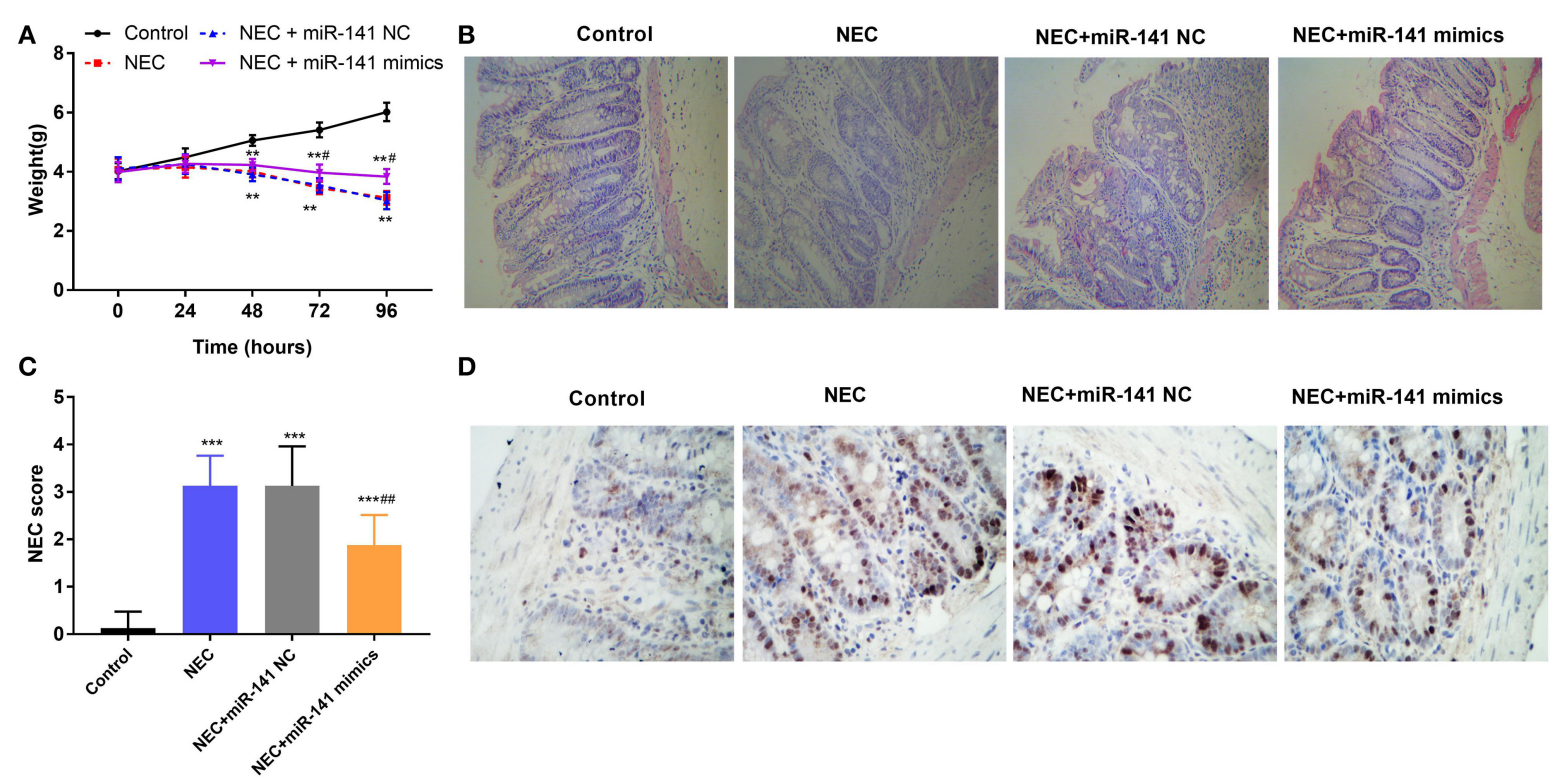
MiR-141-3p eased the damage to intestinal tissues in NEC. After establishment of the NEC model and transfection with miR-141 mimics/miR-141 NC vectors, **(A)** the weight changes of experimental mice (*n* = 8) in different groups were measured; **(B)** the intestinal histopathological changes were observed via hematoxylin and eosin (H&E) staining; **(C)** the intestinal histopathology NEC score was analyzed; **(D)** the cell apoptosis from NEC tissues was assessed through TUNEL assay. **P < 0.01, ***P < 0.001 in comparison with the Control group; ^#^*P* < 0.05, ^*##*^*P* < 0.01 in comparison with the NEC group or NEC+miR-141 NC.

To certify the protection of miR-141-3p for NEC, the weight of neonatal mice was monitored in different groups (Control, NEC, NEC+miR-141 NC, and NEC+miR-141 mimics). We discovered that the weight of neonatal mice was markedly decreased in the NEC group compared with that in the Control group (*P* < 0.01, Figure 2A). After transfection with miR-141-3p mimics, the weight of neonatal mice was clearly recovered when constructing the NEC model for 72 h (*P* < 0.001, Figure 2A). HE staining results displayed that the structure of the intestinal tissues were normal and no inflammatory cell infiltration in the Control group. However, the observed lesions from the NEC group presented the severely necrotic intestinal tissue with the increased numbers of infiltrating inflammatory cells. Surprisingly, overexpression of miR-141-3p significantly improved the damage of intestinal tissue (Figure 2B).

Additionally, the intestinal histopathological score in the NEC group was increased as contrasted with the Control group (*P* < 0.001). Inversely, overexpressed miR-141-3p decreased the NEC score (*P* < 0.001, Figure 2C). Furthermore, TUNEL staining revealed apoptosis of intestinal epithelial cells. Microscopically, we noted that a large number of cell nuclei were blue in the NEC group, which presented strong positive. But partial cell nuclei were blue in the NEC+miR-miR-141 mimics group, which exhibited as positive (Figure 2D). These observations hinted that miR-141-3p distinctly inhibited cell apoptosis. In a word, the above results showcased that miR-141-3p could mitigate the damage to intestinal tissues of NEC.

### miR-141-3p Alleviated Inflammation Response and Oxidative Stress Damage in NEC

**Figure 3:**
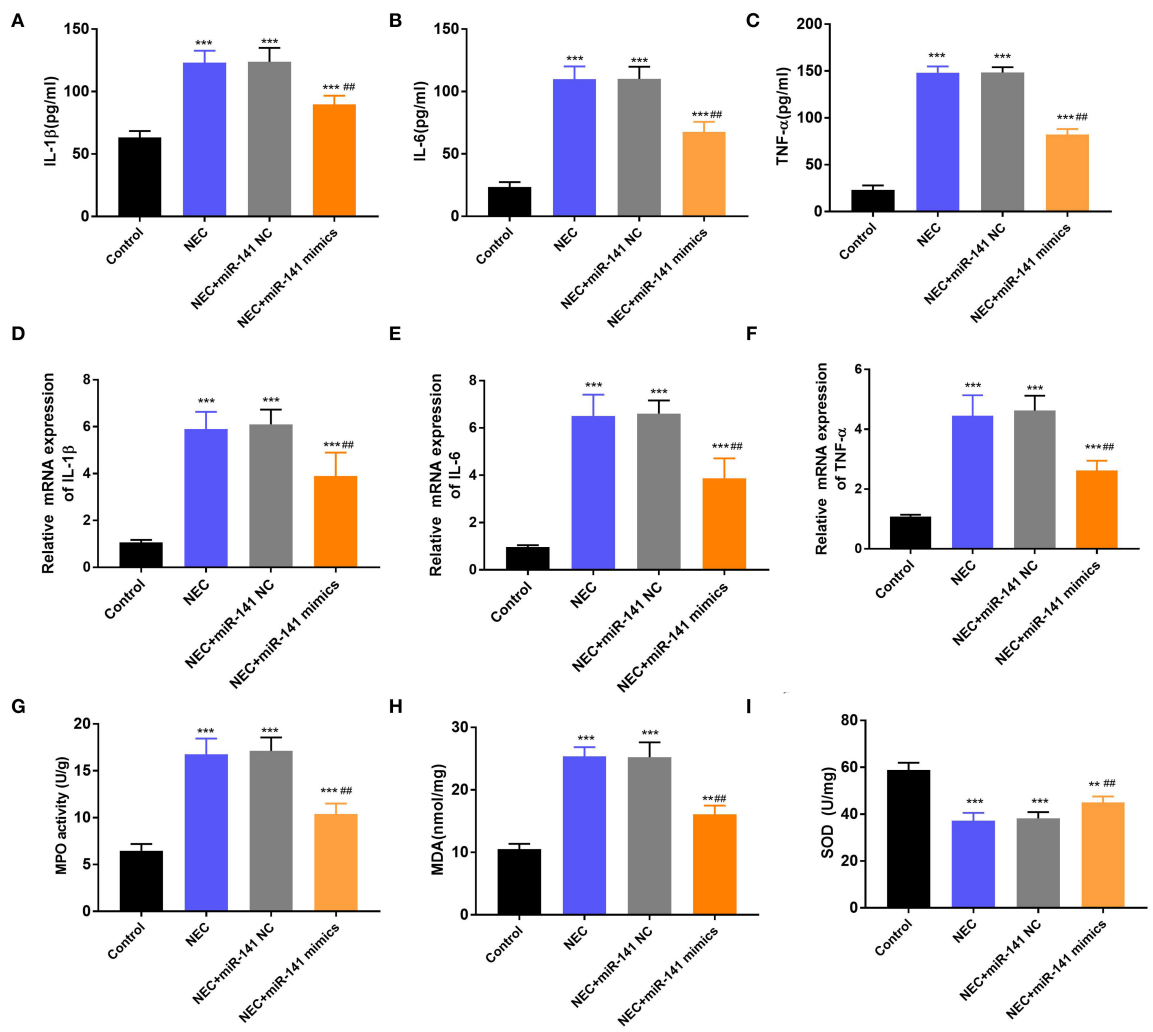
MiR-141-3p regulated the inflammation response and oxidative stress damage in NEC. After establishment of the NEC model and transfection with miR-141 mimics/miR-141 NC vectors, the concentrations of **(A)** IL-1β, **(B)** IL-6, and **(C)** TNF-α were assayed via ELISA; the mRNA expression of **(D)** IL-1β, **(E)** IL-6, and **(F)** TNF-α were examined via RT-qPCR; **(G)** the MPO activity, the levels of **(H)** MDA and **(I)** SOD were evaluated via the corresponding kits. **P < 0.01, ***P < 0.001 in comparison with the Control group; ^##^*P* < 0.01 in comparison with the NEC group or NEC+miR-141 NC.

In the next experiment, we determined the accumulation of inflammatory cytokines (IL-1β, IL-6, and TNF-α) in the Control, NEC, NEC+miR-141 NC, and NEC+miR-141 mimics groups. We observed that the concentrations and mRNA expression of IL-1β, IL-6, and TNF-α were significantly increased in the NEC group (*P* < 0.001, Figures 3A–F). Nevertheless, in the NEC+miR-141 mimics group, the above phenomenon was obviously overturned (*P* < 0.001, Figures 3A–F). Beyond these, compared with the Control group, the levels of MPO and MDA were conspicuously augmented, but the level of SOD was clearly declined in the NEC group (*P* < 0.001, Figures 3G–I). Compared with the NEC+miR-141 NC group, the NEC+miR-141 mimics group prohibited the levels of MPO and MDA and, meanwhile, increased SOD content (*P* < 0.01, Figures 3G–I). The above-involved consequences evinced that miR-141-3p could allay inflammation response and oxidative stress damage in NEC.

### MNX1 Was a Direct Target of miR-141-3p

**Figure 4:**
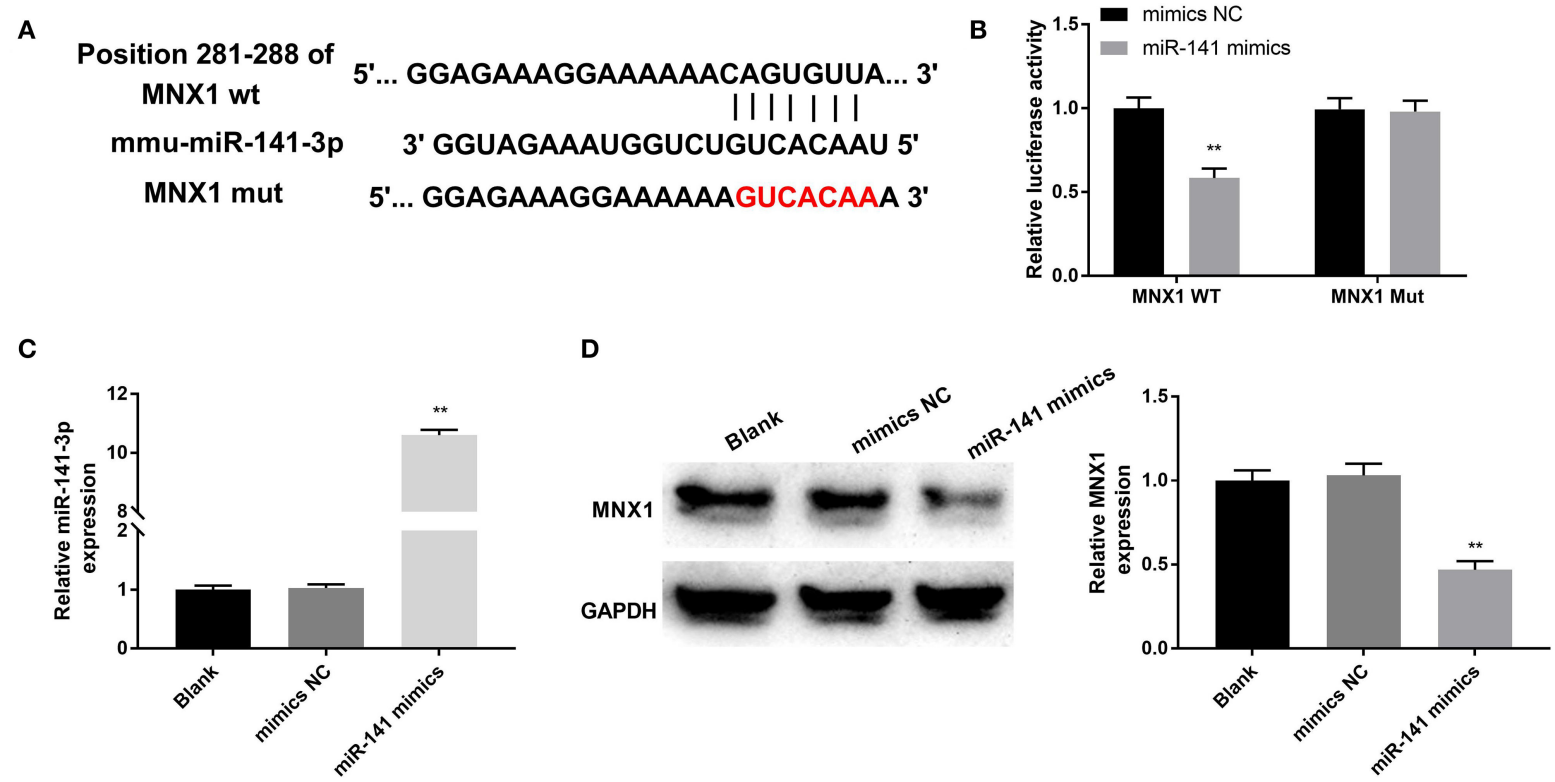
MNX1 was a newfound target of miR-141-3p. **(A)** The sequence relation between miR-141-3p and MNX1 was predicated via Starbase software; **(B)** the luciferase activity was appraised via dual luciferase reporter system; after miR-141 mimics and miR-141 NC vectors transfection, **(C)** the transfection efficiency of miR-141-3p was monitored via RT-qPCR; **(D)** protein level of MNX1 was determined via Western blot assay. **P < 0.01 in comparison with the Blank group.

The relevance between MNX1 and miR-141-3p was forecasted through applying Starbase software and the dual luciferase reporter assay. In Figure 4A, the supposed binding site between MNX1 and miR-141-3p sequences was exhibited. Additionally, the dual luciferase reporter assay revealed that co-transfection with miR-141-3p mimics and MNX1-WT significantly restrained the luciferase activity (*P* < 0.01, Figure 4B). the RT-qPCR assay disclosed the up-expressed miR-141-3p in miR-141-3p mimics transfected cells (*P* < 0.01, Figure 4C). Further, the Western blot assay showcased that the protein level of MNX1 was visibly declined in miR-141-3p mimics transfected cells (*P* < 0.01, Figure 4D). In short, the results explained that MNX1 was a new-predicated target gene of miR-141-3p and negatively regulated by miR-141-3p.

### MNX1 Eliminated the Functions of miR-141-3p in Inflammatory Response of IEC-6 Cells

**Figure 5:**
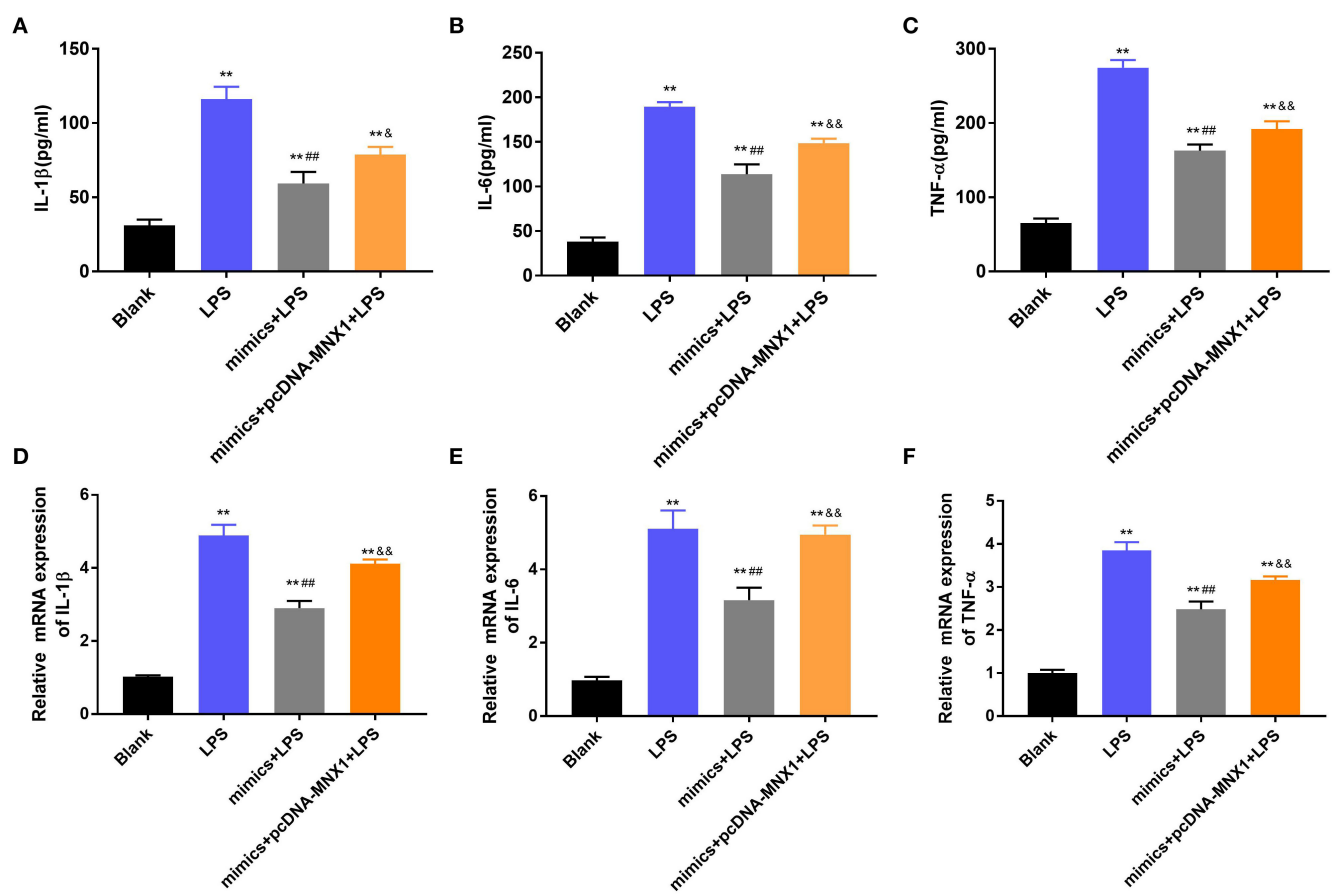
MNX1 eliminated the functions of miR-141-3p in inflammatory response of IEC-6 cells. After LPS irritation and the correlative vectors transfection, the concentrations of **(A)** IL-1β, **(B)** IL-6, and **(C)** TNF-α were examined via ELISA; the mRNA expression of **(D)** IL-1β, **(E)** IL-6, and **(F)** TNF-α were tested via RT-qPCR. **P < 0.01 in comparison with the Blank group; ^##^*P* < 0.01 in comparison with the LPS group; ^&^*P* < 0.05, ^&&^*P* < 0.01 in comparison with mimics+LPS.

To explore whether MNX1 joins in regulating the protective function of miR-141-3p in NEC, the vector of pcDNA-MNX1 was transfected into IEC-6 cells to overexpress MNX1 expression. After LPS irritation, the concentrations and mRNA expression of IL-1β, IL-6, and TNF-α were all significantly upgraded in IEC-6 cells (*P* < 0.01, Figures 5A–F). But the above results were reversed by overexpression of miR-141-3p (*P* < 0.01, Figures 5A–F). When pcDNA-MNX1 was transfected into IEC-6 cells, we surprisingly discovered that the decreased productions of IL-1β, IL-6, and TNF-α triggered by overexpressed miR-141-3p were overturned in LPS-treated IEC-6 cells (*P* < 0.05, *P* < 0.01, Figures 5A–F). These findings revealed that MNX1 could eliminate the protective functions of miR-141-3p in LPS-damaged IEC-6 cells.

### MNX1 Regulated the Functions of miR-141-3p in IEC-6 Cell Growth

**Figure 6:**
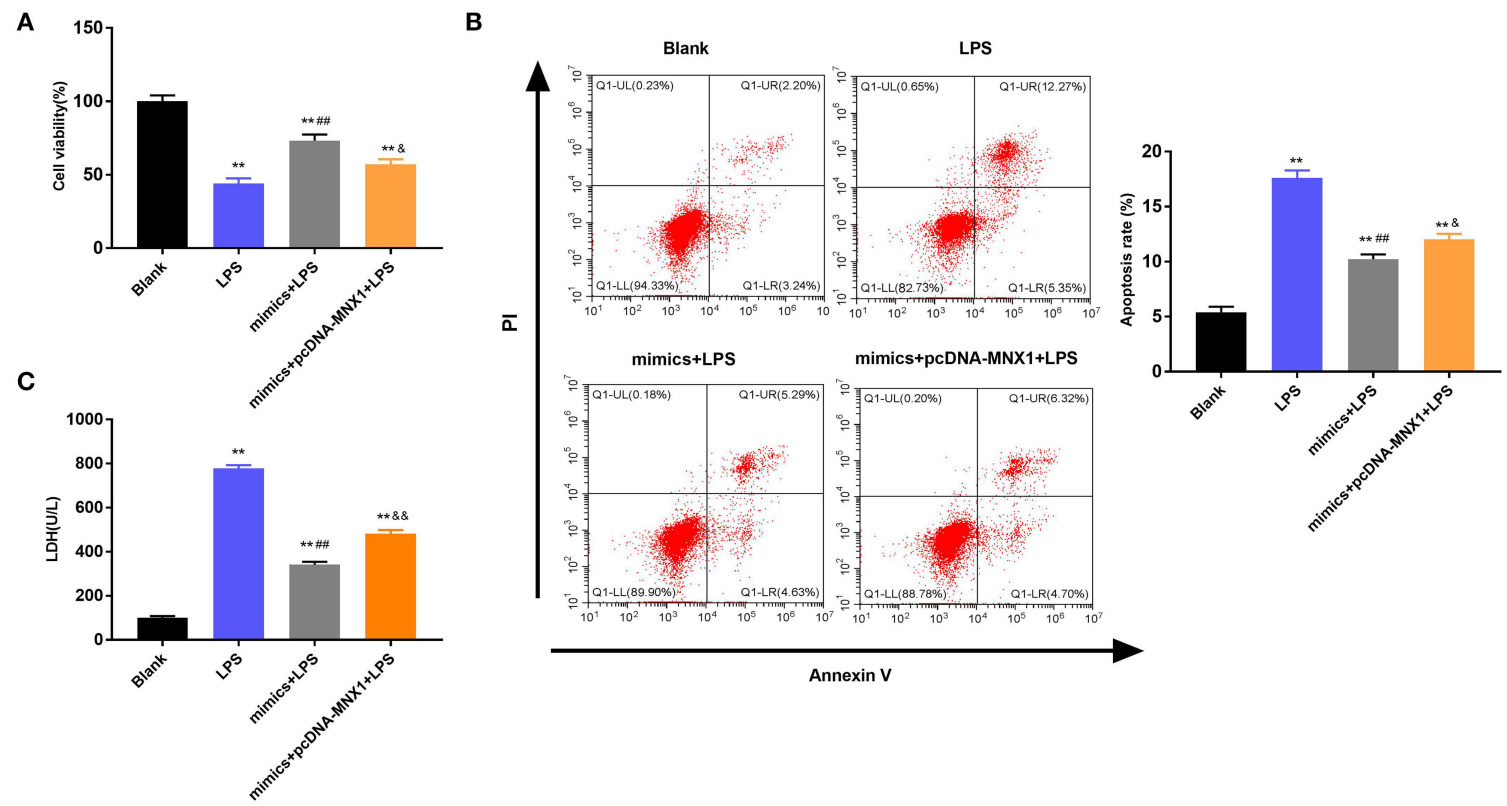
MNX1 modulated the influences of miR-141-3p in IEC-6 cell viability and apoptosis. After LPS irritation and the correlative vectors transfection, **(A)** the vitality of IEC-6 cells was assessed via MTT assay; **(B)** apoptotic degree of IEC-6 cells was evaluated via flow cytometry assay; **(C)** cell death was tested via LDH activity analysis. **P < 0.01 in comparison with the Blank group; ^##^*P* < 0.01 in comparison with the LPS group; ^&^*P* < 0.05, ^&&^*P* < 0.01 in comparison with mimics+LPS.

Further exploration uncovered the regulatory functions of MNX1 in IEC-6 cell viability and apoptosis. The MTT assay explained that LPS irritation clearly restrained cell viability, promoted apoptosis, and increased LDH activity in IEC-6 cells (*P* < 0.01, Figures 6A–C). Nonetheless, overexpressed miR-141-3p expedited cell viability, prohibited apoptosis, and declined LDH activity in LPS-irritated IEC-6 cells (*P* < 0.01, Figures 6A–C). More interestingly, MNX1 overexpression crippled the functions of miR-141-3p in LPS-affected IEC-6 cell viability and apoptosis (*P* < 0.05, *P* < 0.01, Figures 6A–C). All aforesaid consequences imparted that MNX1 subdued the functions of miR-141-3p in IEC-6 cell growth.

## Discussion

NEC is a common acute intestinal inflammatory necrotizing ailment in neonates (24). Recently, studies on the pathogenesis of NEC have primarily focused on the intestinal mucosal barrier and the interaction of intestinal innate immunity and the microbial host (25). Its detailed pathogenesis has not been fully defined. In the existing research, the significant roles of miRNAs in NEC have been reported; however, functions of miR-141-3p in this disease are still ambiguous. Here, we found the aberrant expression of miR-141-3p and MNX1 in NEC tissues. Moreover, the protective functions of miR-141-3p in inflammation response and oxidative stress damage in NEC were certified. Additionally, we found that MNX1 was a target gene of miR-141-3p, and overexpressed MNX1 overturned the influence of miR-141-3p in LPS-damaged IEC-6 cells.

Various studies have discovered that miRNA can play important roles in inflammatory response; innate immunity; tumor formation, proliferation, and differentiation; and apoptosis of intestinal epithelial cells (26, 27). Meanwhile, miRNAs are linked to various intestinal physiological and pathological processes through modulating the intestinal immune or inflammatory response (28). In newborns, miR-146a is reported to downregulate IRAK1 expression to prevent bacteria-triggered intestinal epithelial damage (29). Additionally, miR-23b and miR-150 are discovered to participate in regulating the intestinal inflammatory response (30, 31). As a crucial miRNA, miR-141-3p has been investigated in multifarious ailments (32, 33). Moreover, abnormally expressed miR-141-3p was reported in ulcerative colitis (34). An attractive study disclosed that miR-141-3p lightened inflammation and triggered an anti-inflammatory effect in experimental autoimmune myocarditis mice through suppressing STAT4 expression (35). Additionally, Cheng et al. disclosed that miR-141 could ease ultraviolet (UV)-triggered oxidative stress in human retinal pigment epithelium cells (36). Herein, we observed the low-expressed miR-141-3p in NEC tissues. Further research disclosed that miR-141-3p relieved inflammation response and oxidative stress damage in NEC. These findings hint that miR-141-3p could improve NEC of neonates.

As a vital developmental gene, MNX1 has been investigated in all kinds of cancers, such as colorectal cancer (CRC) (37), bladder cancer (38), and squamous cervical cancer (39), which joins in regulating cell proliferation in these aforementioned cancers. In research involving newborns, MNX1 has been reported in neonatal diabetes, and its expression was clearly upregulated in pancreatic epithelium (15). Similar to this consequence, we observed that MNX1 was conspicuously enriched in NEC tissues. Additionally, the negative relationship between MNX1 and miR-141-3p was testified, and MNX1 was predicated as a neoteric target gene of miR-141-3p. Previous research showcased that miR-141 could assuage LPS-caused WI-38 fibroblast inflammatory injury through ascending NOX2 expression (40). In the next experiment, we also discovered that miR-141-3p could restrain LPS-triggered intestinal epithelial inflammatory cytokine (IL-1β, IL-6, and TNF-α) secretions. Further, MNX1 was found to activate IL-6 expression in Hodgkin lymphoma cells through regulating PI3K signaling (41), which might participate in mediating inflammatory response. In our research, the consequences exhibited that MNX1 overexpression increased IL-1β, IL-6, and TNF-α productions in IEC-6 cells with LPS irritation and miR-141 mimics transfection. These abovementioned experiments revealed that MNX1 could ease the influences of miR-141-3p in inflammatory response of IEC-6 cells.

Numerous studies have certified that miR-141-3p regulates cell proliferation and apoptosis in multiple cancers (42, 43). Moreover, MNX1 has been testified to expedite insulinoma cell proliferation via interaction with NONO protein (44). In the course of inflammation, the capacity of cell viability and apoptosis tend to change naturally (45). LDH activity has been confirmed to be a vital indicator to detect cell apoptosis and cell necrosis (46). Based on this foregoing research, we discovered that overexpressed miR-141-3p clearly accelerates cell viability while it restrained cell apoptosis and LDH activity in LPS-damaged IEC-6 cells. What's more, MNX1 overexpression reversed the functions of miR-141-3p in cell viability and apoptosis. These explorations hinted that MNX1 might regulate the functions of miR-141-3p in LPS-damaged IEC-6 cells.

Taken together, our research reveals that miR-141-3p could improve inflammation response and oxidative stress damage in NEC via targeting MNX1. This conclusion might provide a therapeutic strategy for curing NEC of neonates. Nonetheless, further research is still necessary for expounding the detail mechanisms of miR-141-3p-MNX1 axis in NEC.

## Data Availability Statement

All datasets generated for this study are included in the article.

## Ethics Statement

This study was conducted after obtaining approval of Shenzhen Hospital of Southern Medical University's ethical committee and all experiments were in accordance with the guide for the care and use of laboratory animals established by United States National Institutes of Health (Bethesda, MD, USA).

## Author Contributions

HC: conception and design and analysis of data. HC, LZ, and WZ: drafting the article. XL and BL: revising the article critically for important intellectual content. All authors read and approved the final manuscript.

## Conflict of Interest

The authors declare that the research was conducted in the absence of any commercial or financial relationships that could be construed as a potential conflict of interest.
